# Life course and work ability among older adults: ELSI-Brazil

**DOI:** 10.11606/S1518-8787.2018052000648

**Published:** 2018-10-25

**Authors:** Camila Menezes Sabino de Castro, Maria Fernanda Lima-Costa, Cibele Comini César, Jorge Alexandre Barbosa Neves, Fabíola Bof de Andrade, Paulo Roberto Borges de Souza, Rosana Ferreira Sampaio

**Affiliations:** I Fundação Oswaldo Cruz. Instituto René Rachou. Programa de Pós-graduação em Saúde Coletiva. Belo Horizonte, MG, Brasil; II Universidade Federal de Minas Gerais. Faculdade de Ciências Econômicas. Centro de Desenvolvimento e Planejamento Regional. Belo Horizonte, MG, Brasil; III Universidade Federal de Minas Gerais. Faculdade de Ciências Econômicas. Departamento de Sociologia. Belo Horizonte, MG, Brasil; IV Fundação Oswaldo Cruz. Instituto René Rachou. Núcleo de Estudos em Saúde Pública e Envelhecimento. Belo Horizonte, MG, Brasil; V Fundação Oswaldo Cruz. Instituto de Comunicação e Informação Científica e Tecnológica em Saúde. Rio de Janeiro, RJ, Brasil; VI Universidade Federal de Minas Gerais. Escola de Educação Física, Fisioterapia e Terapia Ocupacional. Programa de Pós-graduação em Ciências da Reabilitação. Belo Horizonte, MG, Brasil

**Keywords:** Aged, Work Capacity Evaluation, Health, Chronic Disease, Socioeconomic Factors, Epidemiologic Factors, Health Surveys, Idoso, Avaliação da Capacidade de Trabalho, Saúde, Doença Crônica, Fatores Socioeconômicos, Fatores Epidemiológicos, Inquéritos Epidemiológicos

## Abstract

**OBJECTIVE:**

To examine factors associated with perception of work ability in a nationally representative sample of Brazilians aged 50 years and over.

**METHODS:**

We used data from 8,903 participants of the baseline survey of the Brazilian Longitudinal Study of Aging (ELSI-Brazil). The dependent variable was self-rated work ability (good or very good *versus* fair, poor, or very poor). Independent variables included factors that operate at the beginning, middle, and current stage of life. Multivariate analysis was based on prevalence ratios (PR) and 95% confidence intervals (95%CI) estimated by Poisson regression.

**RESULTS:**

Good work ability was reported by 49% of \ participants (49.4% among men and 48.6% among women). Results of the multivariate analysis showed that, for both men and women, good work ability showed positive and statistically significant associations (p < 0.05) with good health up to 15 years of age (PR = 1.22 and 1.18 , respectively), educational level ≥ 8 years (PR = 1.19 and 1.21, respectively), and current good self-rated health (PR = 1.88 and 1.94, respectively). Negative associations were observed for current age (PR = 0.99 for each increase of one year among men and women), medical diagnosis of depression (PR = 0.70 for men and PR = 0.87 for women), and having one or more at least chronic diseases (PR = 0.88 for men and 0.91 for women). Only for men, positive associations for the age at which they started working (PR = 1.14 and 1.12 for 11–17 and ≥ 18 years, respectively) and living with a spouse (PR = 1.09) were found.

**CONCLUSIONS:**

Work ability in older ages is built over the life course, particularly by the health conditions in childhood and adolescence, age at which men begin working, educational level, and health conditions in older ages. Policies aimed at increasing longevity in the labor market must take these factors into account.

## RESUMO

**OBJETIVO:**

Examinar os fatores associados à percepção da capacidade para o trabalho em amostra nacional representativa da população brasileira com 50 anos ou mais.

**MÉTODOS:**

Foram utilizados dados de 8.903 participantes da linha de base do Estudo Longitudinal da Saúde dos Idosos Brasileiros (ELSI-Brasil). A variável dependente foi a autoavaliação da capacidade para o trabalho (boa ou muito boa *versus* razoável, ruim ou muito ruim). As variáveis independentes incluíram fatores que operam no início, no meio e na fase atual da vida. A análise multivariada foi baseada em razões de prevalência (RP) e respectivos intervalos de confiança de 95% (IC95%) estimados por meio da regressão de Poisson.

**RESULTADOS:**

A boa capacidade para o trabalho foi informada por 49% dos participantes (49,4% entre homens e 48,6% entre mulheres). Os resultados da análise multivariada mostraram que, tanto para homens quanto para mulheres, a boa capacidade para o trabalho apresentou associações positivas e estatisticamente significantes (p < 0,05) com ter saúde boa até os 15 anos de idade (RP = 1,22 e 1,18, respectivamente), escolaridade ≥ 8 anos (RP = 1,19 e 1,21, respectivamente) e autoavaliação da saúde atual como boa (RP = 1,88 e 1,94, respectivamente). Associações negativas foram observadas para idade atual (RP = 0,99 para cada incremento de um ano), diagnóstico médico de depressão (RP = 0,70 para homens e RP = 0,87 para mulheres) e ter uma ou mais doenças crônicas (RP = 0,88 para homens e 0,91 para mulheres). Apenas entre os homens, associações positivas foram observadas para idade em que começou a trabalhar (RP = 1,14 e 1,12 para 11–17 e ≥ 18 anos) e residência com o cônjuge (RP = 1,09).

**CONCLUSÕES:**

A capacidade para o trabalho nas idades mais velhas é construída ao longo da vida, particularmente pelas condições de saúde na infância e na adolescência, pela idade em que os homens começam a trabalhar, pela escolaridade e pelas condições de saúde nas idades mais velhas. Políticas visando ao aumento da longevidade no mercado de trabalho devem levar em conta esses fatores.

## INTRODUCTION

Aging of the workforce is a global phenomenon. World demographic estimates showed that there were seven persons of active age (20-64 years) for each individual aged 65 years and over in 2015; projections indicate that this ratio will fall to 3.5 in 2050^1^. In addition to this, increased life expectancy means that retired persons use the pension system for a longer time. In Brazil, these changes have been occurring rapidly and they tend to increase. In view of this, a measure adopted by different countries and under discussion in Brazil is the reformulation of social security policies, aiming at extending work life^1–3^. Thus, there is an increasing interest to know the determinants of staying in the work force in older ages.

Good work ability allows older persons to keep working^4^. The work ability demands a balance between personal resources (age, health, skills, values, and attitudes) and working conditions (work environment, demands, and organization)^5^. More recently, this concept has incorporated the family and the close community as factors that can affect work ability in different ways over the life course^6^ ^,^ ^7^. There is a consensus that the ideal balance is dynamic, and changes may occur at different stages of productive life^5^ ^,^ ^8^. A longitudinal study conducted in Finland^9^ has shown that a perception of good or excellent work ability was associated with the age of retirement, which extended the stay in the workforce in approximately six years, and improved quality of life up to five years after leaving the labor market.

Chronological aging compromises the work ability^6^. The first decline occurs approximately at 45 years and the second, at 55^10^. A study conducted with workers in Finland has shown that work ability decreases by approximately 1.5% per year after 45 years of age^11^. Among persons of all ages, health, functional ability, and the characteristics of the work itself are the factors that most affect work ability^4^ ^,^ ^7^ ^,^ ^8^. Research on this subject also indicates that sex inequality worsens the level of work ability among women^12^ ^,^ ^13^.

Throughout life, work ability can vary from excellent to very poor^10^ ^,^ ^1^ ^4^. Epidemiological studies from a life course perspective show that persons are constantly exposed to several factors at different stages of life. These factors can accumulate over time and impact the health and work ability at older ages^15^ ^,^ ^16^.

The few Brazilian studies that have examined the work ability of older persons were restricted to specific occupations^12^ ^,^ ^17^ ^,^ ^18^. The objective of this study was to examine factors associated with the perception of work ability, from a life course perspective, in the Brazilian population aged 50 years and over.

## METHODS

### Data Source and Sample

In this study, we used baseline data from the Brazilian Longitudinal Study of Aging (ELSI-Brazil), whose sample was designed to be representative of the non-institutionalized Brazilian population aged 50 years and over. The baseline data collection was conducted between 2015 and 2016. Sampling used a design with stages of selection, which combined stratification of primary units (municipalities), census tracts, and households. All residents of selected households aged 50 and over were eligible for interview and other procedures. The final sample was estimated at 10,000 persons residents in 70 municipalities from different Brazilian regions (9,412 participated). More details can be found on the homepage of the ELSI-Brazil^a^ and in a previous publication^19^.

### Variables of the Study

The dependent variable of this study was the persons’s perception of their work ability. The information was obtained by the following question: “How do you evaluate your current work ability?” With five possible answers: very good, good, fair, poor, and very poor. For the analysis, the answers were categorized as good (good or very good) and poor (fair, poor, or very poor).

The independent variables were defined following a life course perspective, which considers exposure factors that operate at the beginning, middle, and current stage of life. The variables were temporally divided into distal (related to the characteristics at the beginning of life), intermediate, and proximal (current characteristics). We considered the following distal variables: (1) educational status at the age of 10, whose information was obtained by the question: “When you were 10 years old, did you go to school?”; (2) family financial situation up to 15 years of age, defined by the answer to the question: “Considering your childhood, since your birth to 15 years of age, you would say that your family...”, which had the following response options: above or within average, poor, variable financial situation; (3) health condition up to 15 years of age, which was measured by the question: “Would you say that your health, since your birth to 15 years of age, was...”, which had the following response options f: good (good, very good, or excellent) or poor (fair or poor); and (4) age at which the person started working (10 years or less, 11 to 17 years, and 18 years and over).

Conditions in the middle stages of life were defined by the following variables: (1) physical demands of the work, defined by the answer to the question, “How would you describe the physical demands of the work you had for the most part of your life?”, which was categorized as: never worked or was seated most of the time; was standing or walking, or the work required some physical effort; and the work required intense physical effort; (2) number of complete years of education, categorized as 0–7 years *versus* 8 years or more.

Proximal or current conditions were: (1) age at the date of the interview, (2) position in the household (responsible for the household or not), (3) living arrangements (living or not with a spouse or partner), and (4) current health conditions. Health conditions included self-rated health, defined by the answer to the question, “In general, how would you evaluate your health?”, and chronic diseases, defined by the answer to the question “Has a doctor ever told you that you have “this disease”?” In this analysis, self-rated health was categorized as good (good or very good) or poor (fair, poor, or very poor). The diseases included depression (analyzed separately) and number of chronic diseases, considering hypertension, diabetes, high cholesterol level, cerebrovascular disease, asthma, arthritis or rheumatism, osteoporosis, chronic back pain, cancer, and heart disease. In this analysis, we categorized the answers into none and one or more diseases.

### Data Analysis

**Figure 2:**
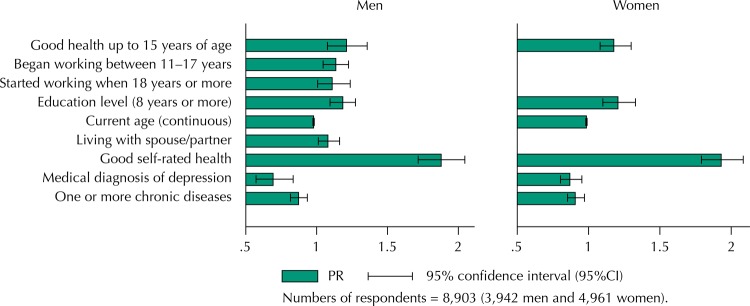
Prevalence ratios (PR) and 95% confidence intervals* of the lifercourse characteristics associated with good self-rated work ability at 50 years and over. Brazilian Longitudinal Study of Aging (ELSI-Brazil), 2015–2016.

In the description of the characteristics of the study participants, the analysis of the differences between men and women was based on Pearson’s chi-square test and Student’s t-test to examine the differences between frequencies and means, respectively. The multivariate analysis was based on prevalence ratios (PR) and respective 95% confidence intervals (95%CI), estimated by robust Poisson regression. We fited the results to illustrate the prevalence ratios of the variables associated with work ability based on the final multivariate model (Figure 2). Collinearity between the study variables was analyzed using the variance inflation factor (VIF < 5). All variables simultaneously entered the multivariate models, since no collinearity was identified.

* Adjusted for all variables listed in the figure, in addition to school situation at the age of 10, family financial situation up to 15 years of age, physical demands of the work, and position in the household.

The unadjusted and multivariate analyses were performed separately for men and women. In all estimates, we used the procedures for complex samples from the statistical program Stata 14.1 (College Station, Texas, USA) in order to consider the effect of the study design and the individuals sample weights.

### Ethical Issues

The ELSI-Brazil was approved by the Research Ethics Committee of the *Fundação Oswaldo Cruz*, Minas Gerais (CAAE 34649814.3.0000.5091). All participants signed the informed consent separately for each research procedure.

## RESULTS

**Figure 1:**
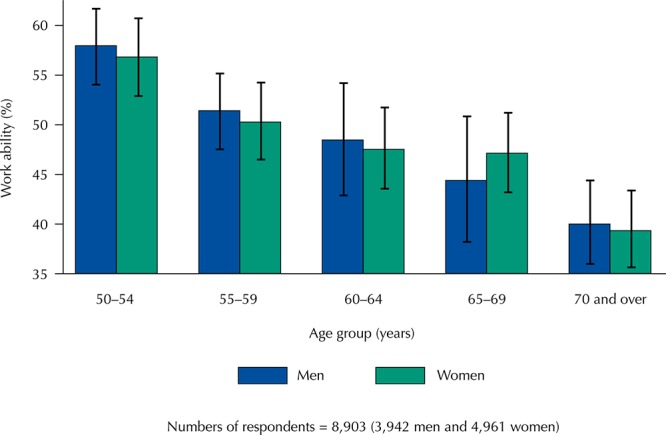
Prevalence of good self-rated work ability according to sex and age in the population aged 50 years and over. Brazilian Longitudinal Study of Aging (ELSI-Brazil), 2015–2016.

Of the 9,412 participants of the baseline of the ELSI-Brazil, 8,903 had complete information for all variables and were included in this analysis. Most participants in this study were women (n = 4,961, 56%). Mean age of the sample was 63.7 years (SD = 9.9), and it was higher among women [64.3 years (SD = 10.0)] than men [62.9 years (SD = 9.8)]. Work ability was evaluated as good by 49.4% of the men and 48.6% of the women. Perception of good work ability decreased with age (Figure 1) both among men (from 57.9% at 50–54 to 51.4% at 55–59 years, 48.5% at 60–64 years, 44.4% at 65–69 years, and 40% at 70 years and over) and among women (56.8%, 50.3%, 47.6%, 47.1%, and 39.4%, respectively).

Table 1 shows the characteristics of the study participants according to sex. Statistically significant differences between men and women were observed for the variables of family financial situation up to 15 years of age, age at which the individual began working, physical demands of the work, age, responsibility for the household, living with a spouse, medical diagnosis of depression, and medical diagnosis of one or more chronic diseases.

**Table 1:** Distribution of good self-rated work ability according with lifecourse characteristics of the population aged 50 years and over, by sex. Brazilian Longitudinal Study of Aging (ELSI-Brazil), 2015–2016.

Variable	Total (n = 8,903)		Men (n = 3,942)		Women (n = 4,961)		p^b^
		
	%^a^	95%CI	%^a^	95%CI	%^a^	95%CI	
Good current work ability	49.0	46.6–51.4	49.4	46.4–52.5	48.6	46.2–51.0	0.511

Early life conditions							

Attended school at the age of 10	75.5	72.3–78.5	75.5	71.6–78.9	75.6	72.4–78.5	0.941
Financial situation of the family up to 15 years of age							
Above or within the average	27.7	25.4–30.0	26.8	24.3–29.4	28.4	25.9–31.1	< 0.001
Poor	57.3	54.9–59.6	56.0	53.2–58.8	58.4	55.7–61.1	
Variable	15.0	13.9–16.3	17.2	15.6–18.9	13.2	12.0–14.4	
Good health up to 15 years of age	78.0	76.2–79.6	78.8	76.8–80.7	77.2	75.2–79.1	0.138
Age at which started working (years)							
< 10	42.2	39.8–44.7	45.3	42.4–48.3	39.5	37.0–42.0	< 0.001
11–17	40.6	38.4–42.8	42.7	40.0–45.5	38.7	36.3–41.2	
≥ 18	17.2	15.2–19.5	12.0	10.4–13.8	21.8	19.2–24.6	

Conditions in the intermediate stages of life							

Physical demands of the previous work							
Never worked/Sitting	17.7	16.1–19.3	16.7	14.9–18.7	18.5	16.6–20.5	< 0.001
Some physical effort	63.4	60.8–66.0	55.9	52.6–59.2	70.1	67.2–72.8	
Intense physical effort	18.9	16.7–21.3	27.6	24.2–30.8	11.4	9.3–13.9	
Education level (full years)							
< 8	63.5	60.5–66.3	62.4	58.8–65.8	64.4	61.4–67.3	0.173

Current life conditions							

Age, mean (SD)	63.7 (9.9)	63.5–63.9	62.9 (9.8)	62.6–63.2	64.3 (10.0)	64.0–64.6	< 0.001
Responsible for the household	73.5	71.7–75.3	87.0	84.7–89.0	61.7	57.8–65.4	< 0.001
Living with spouse or partner	64.3	61.4–67.1	75.6	72.9–78.1	54.3	51.2–57.3	< 0.001
Good self-rated health	44.0	41.5–46.5	43.7	40.4–47.0	44.3	41.7–46.8	0.701
Medical diagnosis of depression	18.6	16.8–20.5	10.6	9.2–12.2	25.6	23.1–28.3	< 0.001
One or more chronic diseases^c^	82.7	81.5–83.9	76.7	75.0–78.3	88.1	86.7–89.4	< 0.001

95%CI: 95% confidence interval.

^a^ Weighted percentage (or mean, when specified) for the sample parameters.

^b^ Pearson’s chi-square test or Student’s t-test with significance level ≤ 0.05, for differences between sexes.

^c^ Medical diagnosis of hypertension, diabetes, cholesterol, cerebrovascular accident, asthma, arthritis or rheumatism, osteoporosis, chronic back pain, cancer, and heart disease.

The results of the multivariate analysis of the association between the characteristics over the life course and work ability according to sex are shown in Table 2 and Figure 2. Among both men and women, good work ability showed positive and statistically significant associations with good health up to 15 years of age (PR = 1.22 and 1.18, respectively), educational level equal to or greater than eight years (PR = 1.19 and 1.21, respectively), and current good self-rated health (PR = 1.88 and 1.94, respectively). In both sexes, negative associations were observed for age (PR = 0.99 for each increase of one year in age in both groups), medical diagnosis of depression (PR = 0.70 for men and PR = 0.87 for women), and having at least one chronic disease (PR = 0.88 and 0.91, respectively). Only among men, good work ability was positively associated with age at which they started working (PR = 1.14 and 1.12 for those who started working at 11–17 years and ^3^ 18 years, respectively) and living with a spouse (PR = 1.09).

**Table 2:** Results of the multivariate analysis of the association between lifecourse characteristics and good self-rated work, by sex. Brazilian Longitudinal Study of Aging (ELSI-Brazil), 2015–2016. (n = 8,903)

Variable	Good current work ability			

	Men (n = 3,942)		Women (n = 4,961)	
	
	%^a^	PR (95%CI)^b^	%^a^	PR (95%CI)^b^
Early life conditions				

Attended school at the age of 10				
No	39.1	1	38.5	1
Yes	52.8	1.04 (0.94–1.15)	51.8	1.06 (0.96–1.16)
Financial situation of the family up to 15 years of age				
Above/Within average	56.3	1	55.1	1
Poor	44.8	0.95 (0.88–1.02)	44.3	0.94 (0.87–1.02)
Variable	53.8	1.02 (0.93–1.12)	53.3	1.02 (0.94–1.11)
Good health up to 15 years of age				
No	36.0	1	37.8	1
Yes	53.1	1.22 (1.08–1.36)	51.8	1.18 (1.08–1.30)
Age at which started working				
≤ 10	41.7	1	42.8	1
11–17	55.4	1.14 (1.05–1.23)	48.8	1.00 (0.93–1.08)
≥ 18	57.5	1.12 (1.01–1.24)	58.7	1.07 (0.96–1.18)

Conditions in the middle stages of life				

Physical demands of the previous work				
Never worked/Sitting	58.1	1	54.4	1
Some physical effort	52.1	0.99 (0.92–1.07)	49.2	1.02 (0.94–1.10)
Intense physical effort	38.6	0.90 (0.81–1.01)	35.6	0.87 (0.73–1.04)
Education level (full years)				
0–7	40.6	1	41.2	1
≥ 8	64.0	1.19 (1.10–1.28)	61.9	1.21 (1.10–1.33)

Current life conditions				

Age (continuous)	61.0	0.99 (0.98–0.99)	61.7	0.99 (0.98–0.99)
Responsibility for the household				
No	46.9	1	50.2	1
Yes	49.8	1.09 (0.95–1.25)	47.6	0.97 (0.88–1.06)
Living with spouse or partner				
No	46.4	1	45.8	1
Yes	50.4	1.09 (1.02–1.17)	50.9	1.05 (0.96–1.15)
Self-rated health				
Poor	32.5	1	32.2	1
Good	71.3	1.88 (1.72–2.05)	69.2	1.94 (1.80–2.09)
Medical diagnosis of depression				
No	51.9	1	51.9	1
Yes	28.6	0.70 (0.58–0.84)	38.8	0.87 (0.80–0.95)
One or more chronic diseases^c^				
0	64.2	1	66.2	1
≥ 1	44.9	0.88 (0.82–0.94)	46.2	0.91 (0.85–0.97)

^a^ Crude percentages considering all sample parameters.

^b^ PR (95%CI): prevalence ratio (95% confidence interval) mutually adjusted for all variables listed in the table and estimated by the Poisson regression model, with robust variance.

^c^ Medical diagnosis of hypertension, diabetes, cholesterol, cerebrovascular accident, asthma, arthritis or rheumatism, osteoporosis, chronic back pain, cancer, and heart disease.

## DISCUSSION

To our knowledge, this is the first nationally representative sample study to examine the influence of experiences over the life course on current work ability. The results show that, for both men and women, good health conditions in early life are associated with better conditions in work ability at older ages. The results also show that education level plays an important role in work ability among older adults, with better conditions among those with higher education. In relation to current conditions, the results highlight the importance of health conditions for work ability, as those who evaluate their current health as poor, who had a medical diagnosis of depression, or who had a medical diagnosis of any other chronic disease had worse work ability.

Health conditions during childhood and adolescence are crucial for the health development of the adult and older adult ^15^. Factors affecting health conditions in childhood and adolescence are associated with participation in school, family income, place of residence, parents’ education and employment, cultural and religious aspects, among others^20^ ^,^ ^21^. Our results reinforce these observations by showing that, among the early life variables considered in this analysis, good health up to 15 years of age presented independent association with work ability at older ages and this association was consistent among men and women.

Another early life characteristic associated with work ability in this analysis was the age at which men began working. The Brazilian legislation allows the work of young persons from the age of 18 years and adolescents from the age of 14 years, allowing as it is carried out as employment, internship, and learning. According to a recent survey by the Brazilian Institute of Geography and Statistics (2017)^22^, at least 190,000 boys and girls up to 13 years of age were part of the illegal group of workers in 2016. Of these children, only 27% had paid work. In relation to the activities, 47.6% were included in agricultural work, 24.7% in the civil construction, transportation, and industry sectors, 21.4% in trade, and 6.3% in domestic services. Our results deepen these observations by showing that the higher the age a men starts working, the greater the tendency to have good work ability. This association was not observed among women. As shown by Kassouf^23^, the earlier the entry into the labor market, the lower the wage, the lower the education level achieved, and the worse the health condition in adult life. Women tend to enter the labor market later and this allows them to acquire a higher education level in relation to men. Mean age of entry into the labor market is lower for men than women, while variance is higher. Therefore, the association between the age when the individual started working and the tendency to have good work ability among older men (but not among women) may be due to both a statistical issue (the larger variance of the age men began working would lead to a greater association) and to the fact that men who enter the labor market earlier have lower income in adult life, and so the association is due to factors not considered in this analysis (such as income received throughout adult life).

The results of this analysis show that men and women with higher education (≥ 8 years) are 20% more likely to evaluate their work ability as good. Workers with lower education levels experience more unfavorable working conditions and, consequently, are in jobs with greater physical demands, which restrict their work ability^4^. We emphasize that education level is the result of accumulated investments throughout life and it is related to personal factors and the characteristics of parents^20^. Thus, from a life-course perspective, we highlight the importance of improving the quantity and quality of education acquired by individuals, particularly among those whose parents have low education levels and who probably belong to the poorest strata of the population.

The decrease in work ability with increasing age, as observed in this study, is expected^4^ ^,^ ^8^ ^,^ ^9^, since older persons generally present worsening health conditions and declining functional ability, as well as difficulties in adapting to changes in work and technological advances^6^ ^,^ ^24^. According to Ilmarinen^13^, in a longitudinal study conducted in Finland, the mean score for the work ability index begins to decrease from 51 to 55 years, and this decrease is consistently observed in the different occupational groups studied. Our results indicate that, among Brazilian older adults, the assessment of work ability decreases by approximately 13%, for both sexes, between the age groups of 50–54 and 55–59 years, and it decreases progressively from the age of 60 years.

Work ability is not separated from life outside work. The family can also affect the work ability of an individual in different ways over the life course. It has become more important to balance work and family life^5^. Our analysis confirms the above observations by showing that living with a spouse or partner is associated with good work ability, but this association was observed only for men. Perhaps this result can be explained by the fact that men can gain health benefits when living with women, since they are more health-oriented. Since the work of Gove^25^, researchers have shown that cohabitation with a spouse of the opposite sex tends to benefit the health of men more than women^26^.

Health is considered as one of the main determinants of work ability^5^ ^,^ ^18^ and self-rated health is a strong predictor of early retirement^27^. All health conditions considered in this analysis presented statistically significant associations with work ability. Among them, self-rated health showed the strongest association. Self-rated health is a multidimensional indicator that expresses the individual’s physical, mental, and social health, and it is a robust predictor of mortality among older adults^28^. We observed negative associations with work ability for the medical diagnosis of chronic diseases and depression, which indicates that persons with these conditions have a worse evaluation of their work ability. Mental disorders are the first cause of disability in the world and the third cause of absenteeism and disability retirement in Brazil^29^. Mental disorders are also significantly associated with unemployment and early retirement because of permanent or temporary incapacity^30^. A longitudinal study conducted for a year in the Netherlands^8^ among individuals aged 45–64 years has shown that workers with chronic diseases have worse work ability compared to their peers without health problems. The major differences between the two groups were found for mental health problems (9.4%) and musculoskeletal problems (4.2%), while heart disease and diabetes mellitus had less impact (2.7% and 2.0% respectively). Our results are in line with these findings as they show a stronger association between depressive symptoms and work ability compared to other chronic diseases. In this case, we cannot rule out reverse causality, since depression may be the cause or the consequence of work incapacity.

This study has advantages and limitations. Among the advantages we can mention the national representative sample of Brazilian older adults. As for the limitations, the collection of self-reported data of the variables related to past experiences may have been influenced by the desire to transmit a socially and culturally acceptable image (bias of social desirability), besides being subject to information bias. Another limitation of the study refers to its cross-sectional nature, which does not allow us to establish temporal associations for the variables related to current conditions. However, it is important to note that longitudinal studies show that age, education level, and current health conditions are associated with current work ability^8^ ^,^ ^9^. Our results are consistent with these observations.

In summary, the results of this study show that work ability in older ages is built over the life course, particularly by the health conditions in childhood and adolescence, age at which men began working, educational level, and health conditions in older ages. Policies aimed at increasing longevity in the labor market must take these factors into account, which profoundly affect the quality of life and work.

## Footnotes

a Fundação Oswaldo Cruz. Brazilian Longitudinal Study of Aging. Rio de Janeiro; c2015 [cited 2017 Nov 28]. Available from: http://elsi.cpqrr.fiocruz.br
